# Modulation of Neutrophil Function by a Secreted Mucinase of *Escherichia coli* O157∶H7

**DOI:** 10.1371/journal.ppat.1000320

**Published:** 2009-02-27

**Authors:** Rose L. Szabady, Mary A. Lokuta, Kevin B. Walters, Anna Huttenlocher, Rodney A. Welch

**Affiliations:** 1 Department of Medical Microbiology and Immunology, School of Medicine and Public Health, University of Wisconsin, Madison, Wisconsin, United States of America; 2 Department of Pediatrics, School of Medicine and Public Health, University of Wisconsin, Madison, Wisconsin, United States of America; University of Washington, United States of America

## Abstract

*Escherichia coli* O157∶H7 is a human enteric pathogen that causes hemorrhagic colitis which can progress to hemolytic uremic syndrome, a severe kidney disease with immune involvement. During infection, *E. coli* O157∶H7 secretes StcE, a metalloprotease that promotes the formation of attaching and effacing lesions and inhibits the complement cascade via cleavage of mucin-type glycoproteins. We found that StcE cleaved the mucin-like, immune cell-restricted glycoproteins CD43 and CD45 on the neutrophil surface and altered neutrophil function. Treatment of human neutrophils with StcE led to increased respiratory burst production and increased cell adhesion. StcE-treated neutrophils exhibited an elongated morphology with defective rear detachment and impaired migration, suggesting that removal of the anti-adhesive capability of CD43 by StcE impairs rear release. Use of zebrafish embryos to model neutrophil migration revealed that StcE induced neutrophil retention in the fin after tissue wounding, suggesting that StcE modulates neutrophil-mediated inflammation *in vivo*. Neutrophils are crucial innate effectors of the antibacterial immune response and can contribute to severe complications caused by infection with *E. coli* O157∶H7. Our data suggest that the StcE mucinase can play an immunomodulatory role by directly altering neutrophil function during infection. StcE may contribute to inflammation and tissue destruction by mediating inappropriate neutrophil adhesion and activation.

## Author Summary

Enterohemorrhagic *Escherichia coli* (EHEC) poses a significant threat to the U.S. food supply, causing foodborne gastrointestinal disease in humans that can progress to hemolytic uremic syndrome (HUS), a potentially fatal kidney disease. Research suggests that EHEC strains are growing more virulent, resulting in a higher incidence of hospitalization and development of HUS from recent produce-associated outbreaks. Although immune dysregulation is a feature of HUS disease, the specific mechanisms contributing to altered immune function require investigation. Furthermore, the contribution of the immune response to early intestinal disease is not known. StcE is a secreted protease of EHEC that is expressed during infection and may contribute to virulence via cleavage of mucin-like glycoproteins. In this study, we define mucinase activity toward glycoproteins on the surface of human neutrophils and find that StcE alters neutrophil activity by interacting with these proteins. StcE affected crucial neutrophil functions including oxidative burst production and migration. The effects of StcE were both cleavage-dependent and cleavage-independent, providing insight into a novel mechanism for mediating neutrophil function via mucin interactions. Our study reports an immune-modulating role for a potential EHEC virulence factor and provides a possible explanation for altered neutrophil phenotypes observed during *E. coli* O157∶H7-induced disease.

## Introduction

Enterohemorrhagic *Escherichia coli* (EHEC) of serogroup O157∶H7 is an emerging human diarrheal pathogen associated with numerous food-borne outbreaks in the U.S. Infection of the colon by EHEC causes mild diarrhea that proceeds to bloody colitis and can be acquired following ingestion of fewer than 100 organisms [1]. In 15% of childhood cases, EHEC gastroenteritis progresses to the more serious hemolytic uremic syndrome (HUS), characterized by red blood cell fragmentation, low platelet count, and acute renal failure. HUS can cause severe kidney damage and have outcomes ranging from full recovery to death [2]. EHEC virulence factors include the locus of enterocyte effacement, which confers the ability to form attaching and effacing lesions, and the phage-encoded Shiga toxin, which causes termination of protein synthesis in the microvascular endothelium leading to cell death and tissue destruction [3]. Efforts to study the pathogenesis of EHEC are complicated by the lack of a suitable animal model that fully recapitulates human disease. Although models of EHEC-induced diarrhea in rabbits or injection of purified components leading to HUS-like symptoms in mice and baboons have been described, no model exists that follows the natural progression from EHEC infection to development of HUS [4]–[6].

The immune response is involved in the development of HUS, but it is less clear how the interaction between bacteria and the host immune system influences early EHEC disease progression. Colonic damage can include hemorrhage and edema within the lamina propria with focal necrosis and neutrophil influx [7], but leukocyte infiltration into the intestinal lumen occurs in only ∼50% of EHEC cases and is rarely severe [2]. Patients who progress to HUS demonstrate clear indicators of an inflammatory response with neutrophil involvement, and increased circulating blood leukocytes are correlated with development of disease [8],[9]. Increased levels of interleukin-8 (IL-8) and complexed elastase are found in the blood [10]–[12]. Children with HUS demonstrate infiltration of monocytes and neutrophils into the kidney glomeruli [13],[14]. The onset of HUS occurs 5–7 days after initiation of diarrhea, and it has been suggested that inappropriate immune cell activation in the gut could lead to renal pathology and explain the lag time in the development of disease [15].

The majority of EHEC isolates in the United States carry the 92 kb pO157 virulence plasmid. Carriage of the plasmid is associated with increased incidence of hemorrhagic colitis, HUS, and colonization of the bovine recto-anal junction mucosa [2],[16],[17]. pO157 encodes StcE (Secreted protease of C1-esterase inhibitor), a type II-secreted, 95 kDa zinc-dependent glycoprotease that is produced during EHEC infection [18],[19]. StcE recognizes O-glycan-induced protein conformations in order to cleave the protein backbone of mucin-type glycoproteins [L. Walters, unpublished, [18],[20]].

Mucins are large glycoproteins that coat numerous surfaces in the body and play important roles in cell-cell interactions within the immune system. CD43 and CD45 are large mucin-type glycoproteins expressed exclusively and abundantly on the surface of nearly all hematopoeitic cells including neutrophils [21],[22]. CD45 is a protein tyrosine phosphatase that can exist as several isoforms which vary primarily in the length of the terminal O-glycosylated portion of the extracellular domain. Dephosphorylation of Src family kinases by CD45 regulates the signaling threshold in T cells [21]. Little is known about CD45 function on neutrophils, but it may modulate chemotaxis and the oxidative burst [23],[24]. CD43 possesses a large extracellular domain that contains 60–80% of its total molecular mass in sialylated O-glycans. Extensive glycosylation causes the protein to assume a rod-like conformation that protrudes ∼45 nm from the cell surface [25]. The combination of steric hindrance, negative charge, and relative abundance on the cell surface provides an anti-adhesive force [26], and CD43-deficient leukocytes demonstrate increased adhesion *in vitro* and *in vivo* [22], [27]–[29]. The intracellular domain of CD43 interacts with cytoskeletal linker proteins, allowing neutrophils and T cells to cluster CD43 at the rear of the cell, or “uropod”, during adhesion and migration. This removes anti-adhesive force from the leading edge to promote adhesion and/or migration, while providing a useful anti-adhesive force at the uropod [30]–[34].

In this study we report the interaction of the StcE protease with CD43 and CD45 on the neutrophil surface. StcE altered neutrophil function via both cleavage-dependent and cleavage-independent effects. Proteolytic activity of StcE led to increased neutrophil oxidative burst production, while binding of StcE was sufficient to increase neutrophil adhesion, leading to impaired migratory capacity. We propose that the interaction between StcE and CD43 prevents the sialoglycoprotein from providing crucial anti-adhesive force, preventing uropod-mediated detachment leading to impaired migration. Oxidative burst production and migration defects leading to increased neutrophil retention could contribute to tissue destruction and inflammation, as well as bacterial evasion of the immune response. Interaction with neutrophil surface mucins by StcE might therefore represent a novel way of dysregulating the immune response during EHEC disease.

## Results

### StcE Binds the Neutrophil Surface Glycoproteins CD43 and CD45

**Figure 1:**
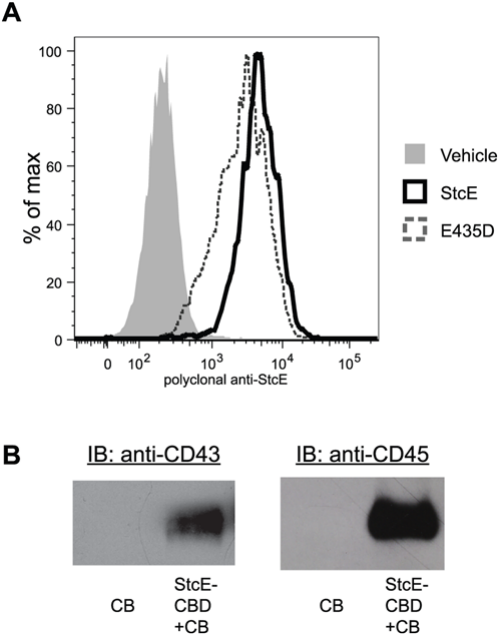
StcE binds to the neutrophil surface and interacts with CD43 and CD45. (A) Neutrophils were incubated with vehicle control, purified StcE protein, or the proteolytically inactive mutant E435D at 1 µg/mL. Cells were stained with polyclonal anti-StcE and analyzed by flow cytometry for StcE binding. (B) StcE fused to a chitin binding domain (StcE-CBD) was bound to chitin beads (CB) and used to precipitate binding partners. dHL-60 cells (5×10^6^) were lysed and incubated with StcE-CBD bound to CB or CB alone. Pulldown reactions were separated by SDS-PAGE and immunoblotted for CD43 (L10) or CD45 (HI-30).

StcE binds to and aggregates cells of the Jurkat leukemic T cell line and binds to the undifferentiated HL-60 promyelocytic cell line [18]. To determine if StcE interacted with neutrophils, primary human neutrophils isolated from whole blood and neutrophil-like differentiated HL-60 cells (dHL-60s) were treated with StcE or a proteolytically inactive StcE point mutant, E435D [18],[35]. Binding was detected by flow cytometry using polyclonal anti-StcE antibody. Both wild-type StcE and E435D bound to neutrophils (Figure 1A) and dHL-60s (data not shown). We next sought to identify the surface determinant responsible for this interaction. The cell-bound mucin-like glycoproteins CD43 (leukosialin) and CD45 (leukocyte common antigen) were previously identified as StcE substrates on Jurkat T cells (unpublished data), and we investigated whether these proteins served as ligands for StcE on the neutrophil surface. As we were unable to immunoprecipitate StcE using available antibodies, we performed direct precipitations with StcE fused to a chitin binding domain (StcE-CBD) and bound to chitin beads (CB). StcE precipitated both CD43 and CD45 from dHL-60 lysates (Figure 1B). Staining of precipitation reactions for total glycoprotein or total protein did not reveal other significant binding partners, suggesting that CD43 and CD45 are the major relevant binding partners on the neutrophil surface (Figure S1).

### StcE Cleaves within the Extracellular Domain of CD43 and CD45

**Figure 2:**
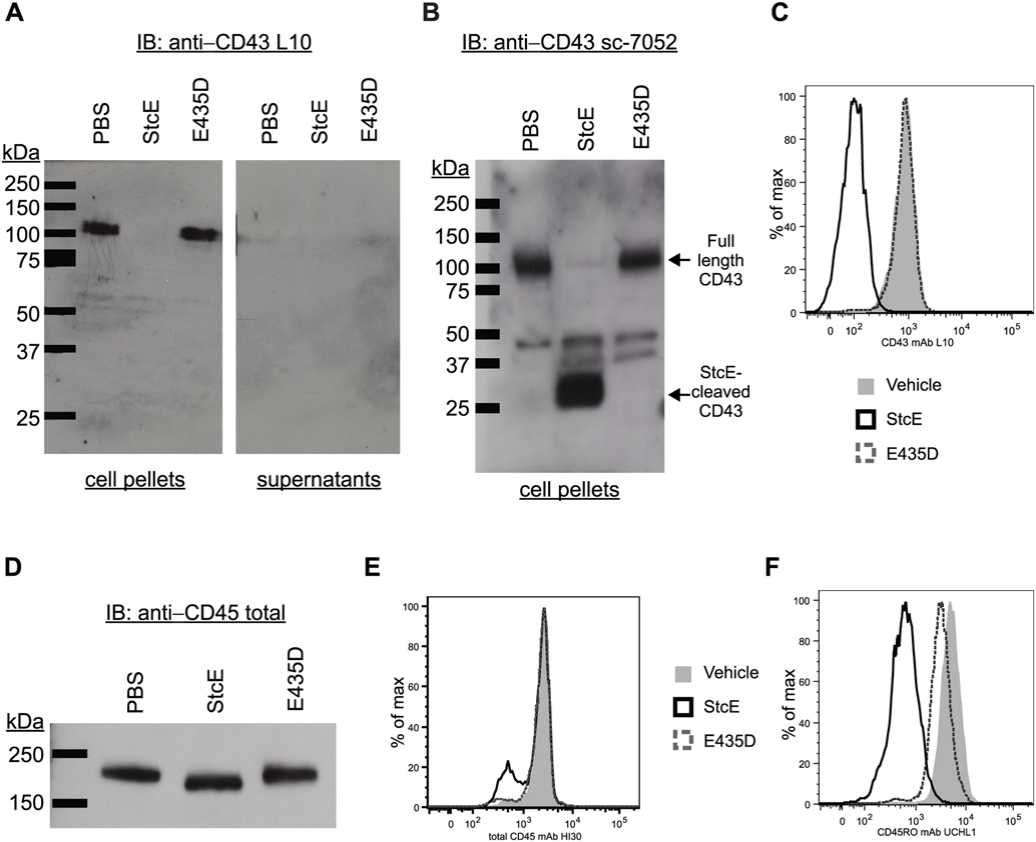
StcE cleaves within the extracellular domain of neutrophil CD43 and CD45. (A) Intact dHL-60s (1×10^6^) were treated with 1 µg/mL purified, endotoxin-free StcE, E435D or vehicle control. Supernatants and cell pellets were individually separated by SDS-PAGE and immunoblotted with L10, which recognizes an extracellular epitope near the N-terminus of CD43. (B) Reactions were performed as in A) and immunoblotted with sc-7052, which recognizes the intracellular C-terminus of CD43. (C) Primary human neutrophils were treated as in (A) and analyzed by flow cytometry to detect the CD43 extracellular domain using PE-labeled L10. (D) Reactions were performed as described in (A) and immunoblotted with HI30, which recognizes all CD45 isoforms. (E) Reactions were performed as in (C) and detected with HI30. F) Reactions were performed as in (C) and detected with UCHL1, which recognizes only the CD45RO isoform.

The pulldown assays described above were performed in the presence of EDTA, which inhibits the metalloprotease activity of StcE. We next investigated whether CD43 and CD45 could be proteolytically cleaved by StcE. Intact dHL-60s were treated with purified, endotoxin-free StcE or proteolytically inactive E435D and analyzed by immunoblottting. Recognition by the anti-CD43 L10 monoclonal antibody (mAb), which recognizes an extracellular epitope near the N-terminus, completely disappeared upon treatment with StcE but not E435D (Figure 2A). An antibody to the intracellular domain of CD43, sc-7052, recognized a StcE-cleaved product of ∼28 kDa (Figure 2B), consistent with a fragment containing the intracellular and transmembrane domains. No L10-reactive cleavage product of any size appeared in the supernatant (Figure 2A), suggesting that the extracellular domain of CD43 was degraded while leaving the intracellular and transmembrane domains intact. Selective cleavage of mucin-like domains is consistent with StcE activity toward other known substrates [18]. Flow cytometric analysis confirmed that presence of the CD43 extracellular epitope recognized by L10 was reduced on the surface of primary human neutrophils following treatment with StcE but not E435D (Figure 2C).

We examined potential cleavage of CD45 using two antibodies. The HI30 mAb recognizes an epitope in the CD45 extracellular domain that is found in all isoforms. Treatment of dHL-60s with StcE resulted in a small size shift from 180 kDa to ∼165 kDa in the band recognized by HI30 (Figure 2D). This suggested that StcE cleaved within the very N-terminal O-glycosylated extracellular portion of CD45. CD45RO is the major isoform present on the neutrophil surface. We next examined cleavage of CD45 using the UCHL1 mAb, which is specific for CD45RO, suggesting that it binds somewhere in the O-glycosylated extracellular region that defines this isoform. Flow cytometric analysis of neutrophils demonstrated no change in staining of total CD45 with HI30, (Figure 2E), while staining of CD45RO with UCHL1 was reduced following StcE treatment (Figure 2F). This suggested that StcE cleaves within the membrane-distal, O-glycosylated extracellular portion of CD45 but does not degrade the protein further.

### Enzymatic Activity of StcE is Required to Increase the Neutrophil Oxidative Burst

**Figure 3:**
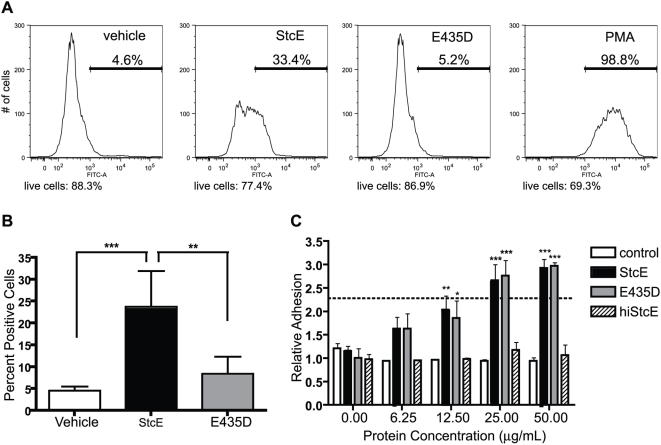
Proteolytic activity of StcE is required to induce the oxidative burst, but not neutrophil adhesion. (A and B) Neutrophils were labeled with dihydrorhodamine 123, treatmed with 5 µg/mL StcE, E435D, or vehicle control, and analyzed by flow cytometry for oxidative burst production as indicated by fluorescence in the FITC channel. PMA served as a positive control. The live cell population was gated by forward and side scatter, and then the “percent positive” gate was drawn to distinguish PBS-treated (negative) and PMA-treated (positive) samples. (A) shows data from a representative experiment, while B) shows the average of five independent experiments. Error bars represent S.D.; *, p<0.05; **, p<0.01 by one-way ANOVA with Bonferroni post test. (C) Neutrophils were fluorescently labeled with calcein-AM, treated with vehicle control, StcE, or E435D at varying concentrations, and allowed to adhere on Fbg for 40 min. Positive control samples were stimulated with 100 nM fMLP for the final 10 min (dotted line). Relative adhesion was calculated by normalizing the number of adherent cells to the average of all vehicle-treated samples. Data shown are mean±S.E.M. of three independent experiments performed in duplicate. *, p<0.05; **, p<0.01; ***, p<0.001 compared to equivalent vehicle control using two-way ANOVA with Bonferroni post test.

CD43 and CD45 are found uniquely on the surface of immune cells and may be important for neutrophil function [21],[22]. To determine if StcE modulates neutrophil function, we examined the effect of StcE treatment on the oxidative burst using a flow-based assay for hydrogen peroxide and superoxide production. Treatment with StcE increased the respiratory burst in the absence of other stimuli (Figure 3A and 3B), while neutrophils treated with proteolytically inactive E435D did not differ significantly from control samples. These findings suggest that StcE modulates neutrophil oxidative function through its protease activity. Both CD43 and CD45 have been suggested to contribute to the neutrophil oxidative burst, and we did not identify the specific mediator of this effect [24],[33].

### StcE Increases Neutrophil Adhesion to the Extracellular Matrix

Because CD43-deficient leukocytes are more adherent, we examined whether removal of the CD43 extracellular domain by StcE altered the ability of human neutrophils to adhere to the extracellular matrix. Treatment with StcE significantly increased neutrophil adhesion on a fibrinogen (Fbg)-coated surface in a dose-dependent manner (Figure 3C). Surprisingly, proteolytic activity of StcE was not required, as binding by E435D induced a similar phenotype. Treatment with either protein resulted in a 2–3 fold increase in adhesion, similar to stimulation with fMLP, which served as a positive control.

As proteolytic activity of StcE was not essential to increase adhesion, we sought to confirm that binding activity of E435D was responsible for this effect. Purified protein was heat-inactivated, which eliminates both binding and proteolysis by StcE and controls for the presence of heat-stable contaminants in the protein preparation. Heat-inactived StcE (hiStcE) had no effect on neutrophil adhesion. E435D is proteolytically inactive but retains substrate binding activity, unlike hiStcE, indicating that binding is the minimal function necessary to induce neutrophil adhesion. To account for the possibility that StcE and E435D increased adhesion by serving as a bridge between CD43 or CD45 and the extracellular matrix, binding of StcE to Fbg was tested by ELISA. StcE alone did not bind appreciably to Fbg (Figure S2), suggesting that effects were specific to interaction of StcE and E435D with the neutrophil surface.

### Relocalization of CD43 is Induced by the Proteolytically Inactive E435D Mutant of StcE

**Figure 4:**
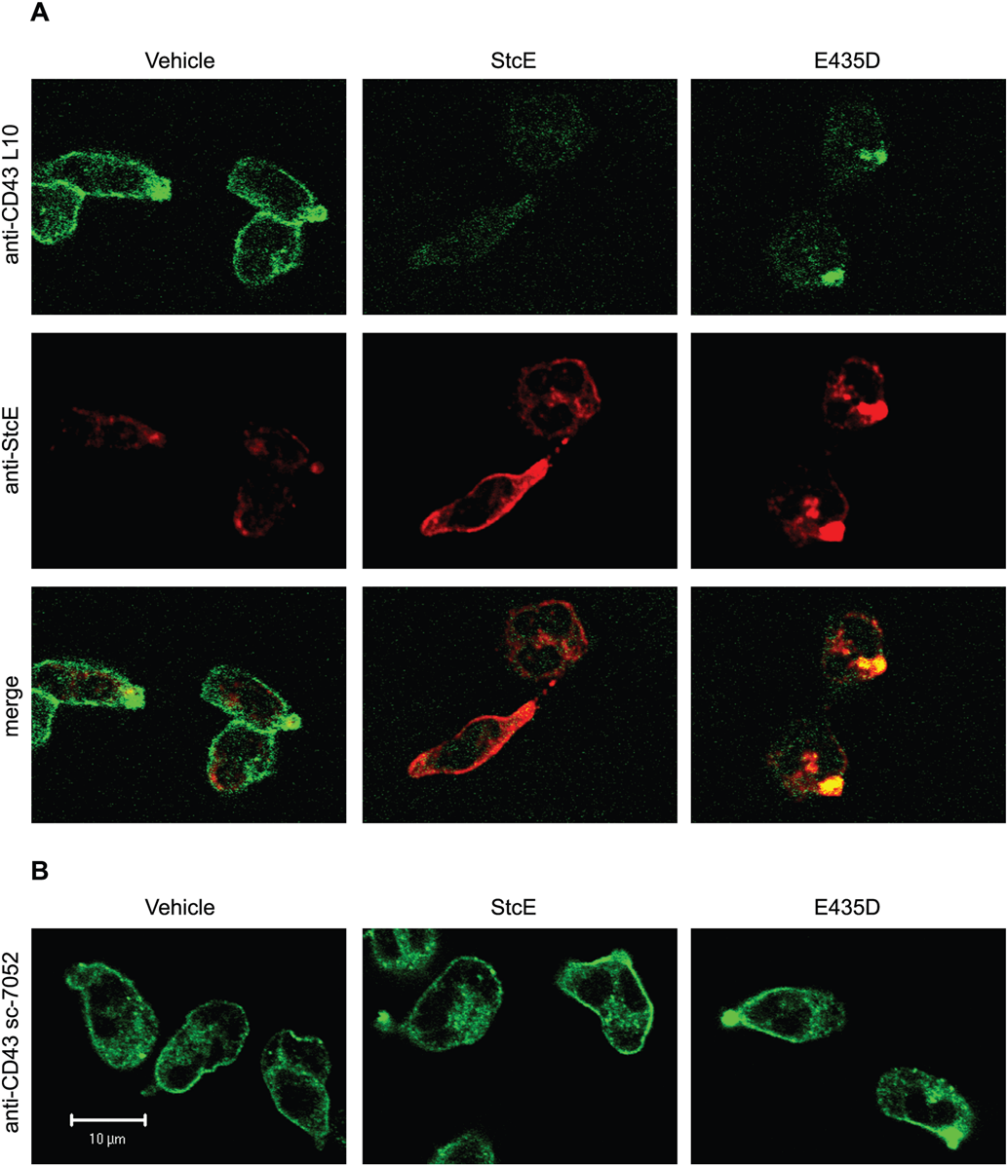
Relocalization of CD43 by the E435D mutant. (A) Neutrophils were treated with 20 µg/mL StcE or E435D for 30 min, followed by 10 min of treatment with 100 nM fMLP to induce firm adhesion and polarization. Cells were fixed and stained with rabbit anti-StcE and mouse anti-CD43 L10 and analyzed by confocal immunofluorescence microscopy. (B) Neutrophils were treated as in (A) and stained for the CD43 intracellular domain with sc-7052. Scale bar represents 10 µm for all images.

CD43 is localized to the uropod during neutrophil adhesion and migration, removing its anti-adhesive force from the front of the cell [30]. The observation that both StcE and E435D induced neutrophil adhesion led us to hypothesize that protein binding to CD43 could interfere with its anti-adhesive function even in the absence of cleavage. This hypothesis is consistent with reports that antibodies to CD43 can induce clustering of the protein at the uropod and increase cell adhesion [30],[32]. We therefore examined how treatment with StcE and E435D affected CD43 localization in adherent neutrophils via confocal immunofluorescence microscopy. Although the anti-StcE antibody exhibited some background staining of vehicle-treated cells, we observed specific and diffuse surface staining of bound StcE (Figure 4A), consistent with flow cytometry data. StcE-treated neutrophils demonstrated reduced membrane staining for the CD43 extracellular domain with L10, consistent with results of immunoblotting and flow cytometry. No change in localization of the CD43 intracellular domain as detected by sc-7052 was observed, confirming that it remained intact and membrane-associated (Figure 4B). In contrast, treatment of neutrophils with E435D caused readily observable relocalization of CD43. Both extracellular and intracellular staining revealed clustering of CD43, and E435D staining was co-localized with the CD43 extracellular domain (Figure 4). Immunofluorescence staining for total CD45 revealed no change in localization induced by StcE or E435D (data not shown).

These data suggest that E435D promotes neutrophil adhesion by clustering CD43 to the uropod and removing its anti-adhesive force from the rest of the cell membrane. To further confirm that E435D bound to native CD43 on the neutrophil surface, we used flow cytometry to investigate the ability of E435D to compete with the L10 mAb for binding to the extracellular domain. Increasing concentrations of E435D led to decreased L10 staining (Figure S3), indicating that E435D bound to CD43 and blocked antibody accessibility. Reduction of L10 binding by StcE was evident at a much lower protein concentration, demonstrating that cleavage by StcE was more efficient than blocking with E435D in preventing L10 antibody binding. Together these findings suggest that while StcE causes loss of CD43 from the neutrophil surface, E435D clusters CD43 in the uropod, leading to removal of anti-adhesive force from the cell membrane and providing an alternative mechanism by which binding alone can induce neutrophil adhesion.

### StcE Impairs Neutrophil Migration *in vitro*

**Figure 5:**
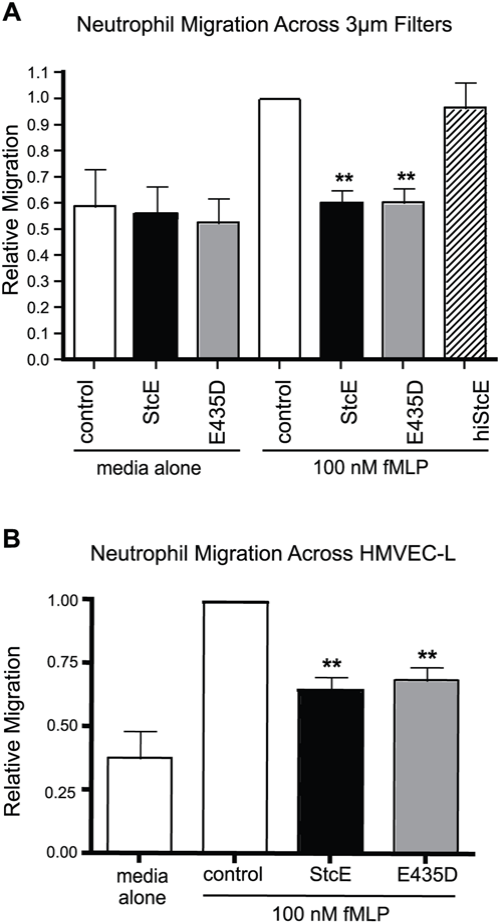
StcE inhibits neutrophil chemotaxis. (A) Neutrophils labeled as in figure 3C were treated with 25 µg/mL StcE or E435D in the upper chamber of Fbg-coated transwell inserts, and media with or without chemoattractant (100 nM fMLP) placed in the lower chamber. Cells were allowed to migrate for 210 min and neutrophils in the lower chamber were quantified and normalized as described. Data shown are mean±S.E.M. of three independent experiments. **, p<0.01 compared to vehicle with chemoattractant by one-way ANOVA using Dunnett post test. (B) Experiments were performed as described in (A), with 50 µg/mL protein and monolayers of HMVEC-L grown on collagen-coated transwell inserts. Data are mean±S.E.M. from six independent experiments analyzed as in (A).

Neutrophil adhesion regulates the development of cell polarity and is required for cells to become migration-competent, but too much adhesion can interfere with migration [36]. We examined how the StcE-induced increase in adhesion affected migratory capabilities of human neutrophils using transwell assays. In the absence of chemoattractant, StcE treatment had no effect on migration across transwell inserts (Figure 5A). In the presence of fMLP as a chemoattractant in the lower chamber, treatment with StcE or E435D caused a significant, 1.7-fold reduction in migration across the filters. Consistent with the results of adhesion experiments, binding but not proteolytic activity of StcE was required to inhibit neutrophil migration, and heat-inactivated protein had no effect.

Circulating leukocytes that detect chemotactic signals first adhere to the vasculature and then transmigrate across the endothelium in order to reach effector sites. To verify that results obtained with purified Fbg extended to interactions with the endothelium, migration experiments were conducted using monolayers of primary human lung microvascular endothelial cells (HMVEC-L). Both StcE- and E435D-treated neutrophils demonstrated decreased migration across HMVEC-L toward fMLP (Figure 5B). These findings confirmed that the defect in neutrophil migration results from the action of StcE on the neutrophil surface and is not specific to the migratory barrier.

**Figure 6:**
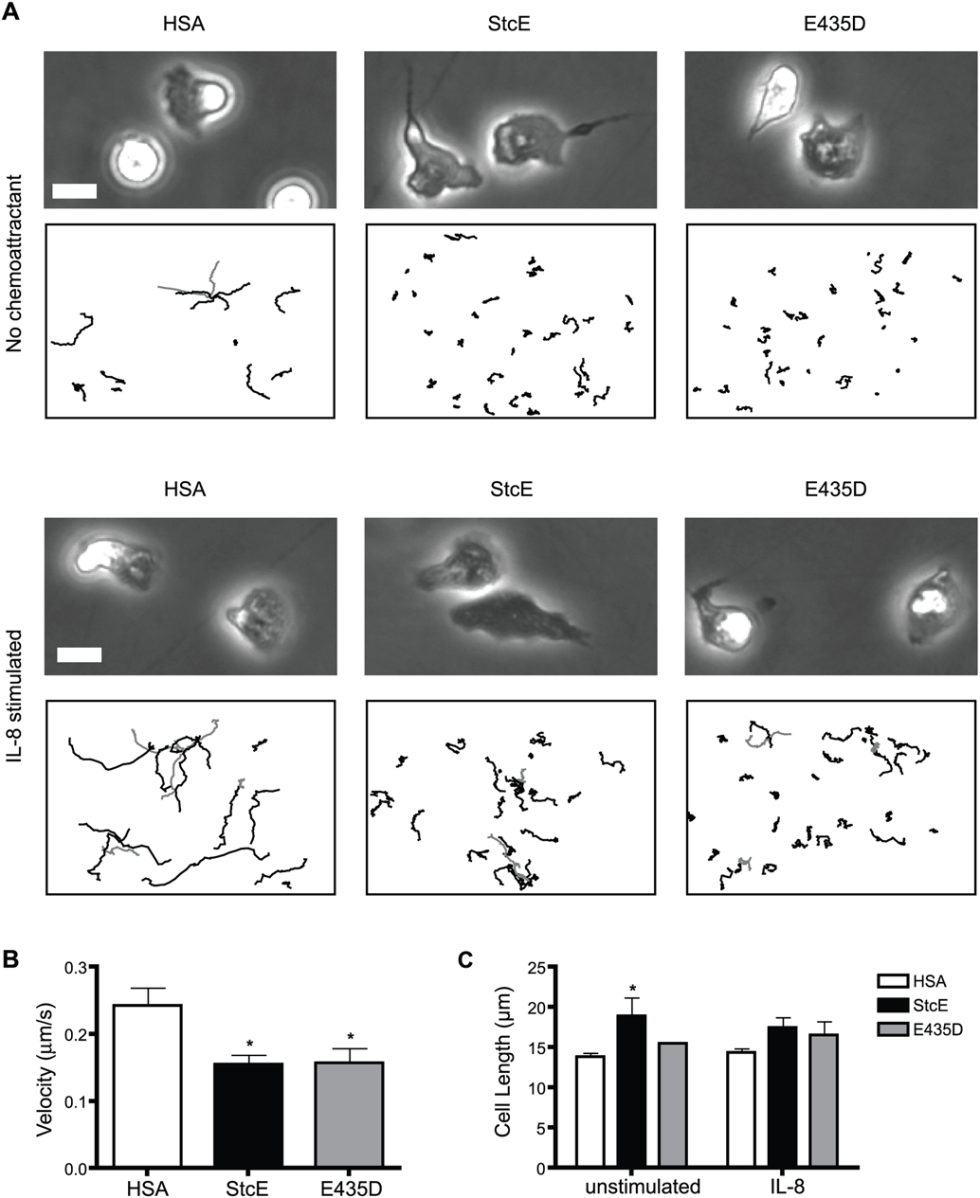
StcE decreases neutrophil random migration via impaired uropod retraction. Neutrophils were treated with human serum albumin (HSA), StcE, or E435D (12.5 µg/ml) in Fbg-coated dishes for 30 min and stimulated with IL-8 for the final 5 min where indicated. Neutrophil migration was imaged by time lapse video microscopy over 10 min. Still images were captured and compiled into videos using Metamorph software (see Videos S1, S2, S3, S4, S5, and S6), and image tracking was performed. (A) Still images from a representative experiment are shown with scale bar representing 10 µm. Graphs of cell tracks below still images indicate individual paths of all cells tracked for each condition over 10 min. (B) Cell velocity was calculated using MetaMorph software for IL-8 stimulated samples. Data are presented as mean±S.E.M. of four independent experiments. *, p<0.05 as compared to HSA by one-way ANOVA with Dunnett's post test. (C) Quantitation of cell length in stimulated and unstimulated samples was performed using Metamorph by measuring the length of all cells in two randomly chosen frames per sample. Data are shown as mean±S.E.M of three independent experiments; *, p<0.05 compared to appropriate HSA control by two-way ANOVA with Bonferroni's post test.

We further examined the effect of StcE on neutrophil migration using time lapse microscopy. The adhesion and migration experiments described above were conducted with PBS as a vehicle control. Although the experimental media contained 5% serum, the formal possibility remained that increased total protein concentration was responsible for the effect of StcE or E435D addition. We therefore evaluated migration in the presence of an equivalent concentration of human serum albumin (HSA), and cell behavior was identical to vehicle treatment (data not shown and Video S1). Neutrophils were treated with HSA, StcE or E435D on a Fbg-coated surface and non-directional migration was imaged in the absence or presence of interleukin-8 (IL-8). Consistent with adhesion and transmigration assays, StcE-treated and E435D-treated neutrophils demonstrated increased adhesion and were visibly impaired in their migratory capabilities (Figure 6A and Videos S2 and S3). IL-8 treatment caused an increase in random migration of neutrophils (Video S4), and both StcE and E435D reduced migration even in the presence of IL-8 (Figure 6A and Videos S5 and S6) and fMLP (data not shown). Quantitation of neutrophil migration was performed on IL-8 treated samples, as these had comparable numbers of adherent cells for control and experimental conditions. Treatment with StcE or E435D significantly reduced neutrophil migration velocity compared to HSA control (Figure 6B). Although they traveled shorter distances over time, StcE- and E435D-treated neutrophils did not appear deficient in production of forward protrusions. However, cells seemed unable to retract their rearward edge and move forward, suggesting that StcE interfered with neutrophil migration by preventing de-adhesion of the uropod. During migration, StcE-treated neutrophils displayed striking morphological differences, with formation of elongated tails at the uropod (Figure 6B). E435D treatment also resulted in elongated morphology, but the phenotype was not as severe. Analysis of cell length confirmed that unstimulated, StcE-treated neutrophils were significantly longer than control cells, while E435D treatment did not cause a significant difference in cell length (Figure 6C). Both StcE-treated and E435D-treated neutrophils exhibited increased cell length in the presence of IL-8, although the increase was not significant compared to IL-8 stimulated controls (Figure 6C). Together our findings suggest that both StcE and E435D interfere with CD43-based anti-adhesion to alter adhesion and migration, but do so via different mechanisms dependent on binding and cleavage of cell surface mucins (StcE) or binding alone (E435D).

### StcE Induces an Inflammation-Like Phenotype during Wound Healing in Zebrafish

**Figure 7:**
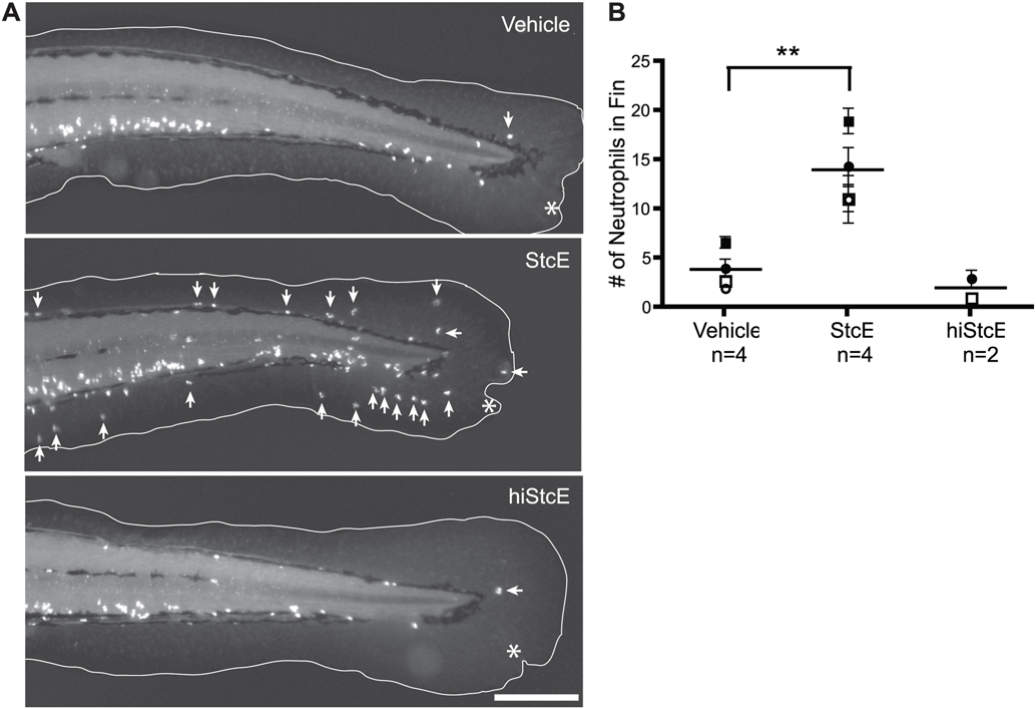
StcE induces mislocalization of zebrafish neutrophils. (A) Zebrafish embryos at 3 dpf were wounded in the ventral fin in the presence of 25 µg/mL StcE, hiStcE, or vehicle control and fixed after 24 hours for whole-mount immunofluorescence with anti-mpo staining. Arrows indicate abnormal localization of neutrophils in the fin of StcE-treated zebrafish. The edge of the fin is outlined in white, and the site of the wound is indicated by an asterisk. The scale bar represents 200 µm. (B) Images from independent experiments were blinded and the number of neutrophils in the fin counted. Data are shown as means of individual experiments (shapes)±S.E.M. and total combined mean (bars); **, p<0.01 of combined means by one-way ANOVA with Dunnett's post test.

Zebrafish share many features of the mammalian immune system and have recently been utilized as a model to study neutrophil migration and chronic inflammation [37]–[39]. We took advantage of the zebrafish model to examine the effects of StcE on neutrophil-mediated inflammation *in vivo*. Zebrafish embryos at 3 days post-fertilization (dpf) were wounded in the ventral tail fin in the presence of a bath of StcE protein. At this stage in development, neutrophils are normally located in the caudal hematopoeitic tissue and circulating in the bloodstream. Wounding of the tail fin induces neutrophil recruitment to the wound, and resolution of this response is generally observed after 24 hours [37]. Wounded embryos were fixed after six or 24 hours and localization of myeloperoxidase (mpo), a neutrophil-specific marker, was examined by immunofluorescence microscopy. Differences in neutrophil recruitment were not observed six hours after wounding (data not shown). At 24 hours, treatment with StcE caused visible neutrophil mislocalization (Figure 7A), resembling chronic inflammation recently observed in zebrafish mutants [39]. Neutrophil mislocalization was quantified by counting neutrophils present in the fin, confirming that treatment with StcE caused a significant increase in number of mislocalized neutrophils (Figure 7B). HiStcE had no effect on neutrophil localization. The inflammation-like phenotype observed in zebrafish embryos suggests that StcE may affect neutrophil motility and trafficking *in vivo* to regulate inflammatory responses.

## Discussion

In this study, we report cleavage of CD43 and CD45 on the human neutrophil surface by StcE, a secreted glycoprotease of *E. coli* O157∶H7. We found that StcE exerted both cleavage-dependent and cleavage-independent effects on neutrophil migration and activation, suggesting that it may modulate the immune response during infection. We have previously reported specific cleavage of mucin-type O-glycoproteins by StcE [18],[20]. CD43 and CD45 share characteristics of these substrates and were the major StcE ligands on the neutrophil surface. We found that StcE cleaved specifically within the O-glycosylated domains of these proteins. The majority of the CD43 extracellular domain is heavily O-glycosylated, and the resultant negative charge and steric hindrance serve to inhibit non-specific cell-cell interactions. StcE degraded this domain, leading to loss of anti-adhesive function. In contrast, the majority of the CD45 extracellular domain is N-glycosylated, and the N-terminal portion, which varies in length by differential splicing, is O-glycosylated. StcE cleaved within this terminal portion, leaving the majority of the protein intact. It is not known what effect the terminal O-glycosylated region has on function of the intracellular phosphatase domain, but cleavage of this region by StcE could promote inhibitory dimerization.

In order to fight infection, neutrophils must leave the bloodstream to reach effector sites. Cells first adhere to the vascular endothelium and then migrate across the endothelial cell layer and through the tissues by sensing and responding to chemoattractant gradients [40]. Adhesion is required to initiate this process, but excessive adhesion can inhibit migration [36]. Neutrophils counter the anti-adhesive function of CD43 by shedding it from their surface when they become activated [34],[41]. The remaining surface-associated CD43 is redistributed to the uropod at the cell rear [30],[31],[42], where it may provide useful anti-adhesive force. Treatment with StcE led to increased neutrophil adhesion that interfered with random migration as well as chemotaxis across filters and endothelial monolayers. Surprisingly, this effect was cleavage-independent. The proteolytically inactive mutant, E435D, caused similar effects to StcE, suggesting that binding was the minimal function required. Our data suggest that removal of CD43 anti-adhesion is the mechanism by which StcE and E435D interfere with migration, although we cannot rule out a role for CD45. StcE degraded the extracellular domain of CD43, while E435D clustered CD43 at the uropod and blocked antibody binding to the extracellular domain. Cleavage of CD43 by StcE reduces anti-adhesive force at the promigratory leading edge, but also relieves the anti-adhesive force in the uropod that could promote rear detachment. E435D, like crosslinking antibodies, induces CD43 relocalization to the uropod, reducing anti-adhesive force at the leading edge. Binding of E435D may also mask the negative charge of CD43 and interfere with anti-adhesion at the rear, causing a similar outcome to StcE via a slightly different mechanism. Increased adhesion can be induced by crosslinking antibodies to CD43 [30], and it is unclear if this results from simple masking of the protein or because antibody binding transduces a pro-adhesive signal. Our results support the hypothesis that the anti-adhesive function of CD43 at the uropod is an important component of cellular migration.

Although it has been proposed that CD45 may regulate chemotactic signaling in neutrophils [23], only recently has it been suggested that CD45 may be directly important for cell adhesion. Shivtiel and colleagues found that bone marrow mononuclear cells deficient in CD45 were more adherent to fibronectin as a result of increased activation of β1 integrins [43]. It is possible that CD45 signaling may be important for neutrophil adhesion and that interaction with CD45 contributes to StcE-mediated effects on adhesion and migration. It is unknown whether StcE cleavage of the terminal O-glycosylated portion of CD45 will affect its signaling capacity. Investigation of the effect of StcE on CD45 signaling is a potential topic for future study.

The observation of a cleavage-independent function for StcE in neutrophils parallels findings with C1-esterase inhibitor (C1-INH), another StcE substrate. StcE-cleaved C1-INH retains its ability to inhibit the complement cascade, and E435D is equally capable of potentiating C1-INH function. StcE binds to C1-INH and the bacterial surface simultaneously, increasing the local concentration of C1-INH and protecting the cell from complement-mediated lysis [35]. If binding of StcE to its substrates is sufficient, what is the purpose of proteolytic activity? Proteolysis may be more important for some substrates than others. For example, cleavage of intestinal mucins might be required to promote colonization, whereas only binding is necessary to affect activity of C1-INH and CD43. Alternatively, proteolytic activity of StcE might be dispensable for its interaction with all substrates but provide enhanced turnover. StcE displays high affinity and low turnover of C1-INH and MUC7 [44], and it is possible that proteolysis provides a mechanism for it to detach from one substrate molecule in order to bind another. This would facilitate interaction of the same StcE protein molecule with different substrates, allowing it to interact with multiple glycoproteins during infection.

Proteolytic activity was not completely dispensable to modulate neutrophil function, as the activities of E435D and StcE were not identical. Active StcE more potently induced an elongated cell morphology during neutrophil migration than did E435D, suggesting that cleavage was more efficient than binding in preventing rear detachment. This could be explained if binding of E435D to CD43 blocked charge repulsion, but removal of the CD43 extracellular domain by StcE eliminated both charge repulsion and steric hindrance. Furthermore, StcE exerted a cleavage-dependent effect on neutrophil oxidative burst production. The specific substrate mediating these effects was not identified, but CD45 is an attractive candidate because it has previously been reported to modulate production of the oxidative burst [24]. CD43 may also play a role in oxidative burst production, although evidence for this is less clear [33]. Regardless of the exact mechanism, the finding that StcE increased the oxidative burst in a cleavage-dependent manner provides further evidence that it may play an immunomodulatory role during infection.

Neutrophils are the first responders to bacterial infection, and pathogens have strategies to combat this response that include inhibition of chemoattractant receptors and inactivation of C5a and IL-8 [45]. To our knowledge, proteolysis of cell surface mucins by StcE represents a novel mechanism for altering neutrophil function. Respiratory burst production and the ability to migrate are crucial capabilities of neutrophils in fighting infection, and alteration of these functions by StcE may lead to a dysregulated immune response during EHEC infection. Neutrophils isolated from children with HUS are more adherent to the vascular endothelium [46], and StcE could contribute to this phenotype. The effect of StcE on neutrophil migration was both rapid and persistent. Increased adhesion and impaired migration were evident after 30 minutes of StcE treatment. After 210 minutes, fewer StcE-treated neutrophils had migrated across transwell filters, suggesting this was not a transient defect that could be overcome with time. The longevity of the effect further suggests that the interaction between StcE and neutrophils may be physiologically relevant. Whether alteration of neutrophil function leads to pro- or anti-inflammatory outcomes may depend on the site of activity and presence of other stimuli. Neutrophils that remain stuck to the endothelium may be unable to migrate into the intestine in response to infection, and StcE may thus protect the bacteria from clearance by the host immune response. The observed decrease in neutrophil migration across endothelial cell monolayers supports the conclusion that StcE could impair migration out of the vasculature and into the intestine. Inhibition of complement activation at sites of infection by StcE-localized C1-INH would reduce production of the chemotactic C5a fragment, further contributing to a migration defect. Alternatively, neutrophils that are stuck to the endothelium may contribute to inflammation and tissue destruction, as seen in the kidneys during HUS. Zebrafish treated with StcE exhibited neutrophil mislocalization to the tissues that resembled recently described inflammatory mutants, lending credence to this hypothesis. The fact that mislocalization occurred at a late but not early time point after wounding may be explained by accumulation of inflammatory signals from neutrophils that are retained in the tissues, leading to progressive neutrophil infiltration and retention over time. Enhancement of the neutrophil oxidative burst by StcE could further contribute to inflammatory tissue damage in the intestines and during HUS as a result of inappropriate neutrophil retention.

The pO157 plasmid is associated with EHEC disease incidence and severity, suggesting that plasmid-encoded genes might contribute to pathogenesis [2],[3]. The plasmid-encoded StcE protein has a dedicated type II secretion system, is co-regulated with known virulence factors, and is produced in detectable amounts during infection, making it a likely virulence candidate. In a recently described disease model of EHEC in rabbits, mutation of the type II secretion system led to decreased colonization, and the authors conclude that lack of StcE secretion might contribute to this defective colonization [47]. Potential contributions of StcE to virulence including inhibition of complement-mediated lysis and promotion of pedestal formation have been described [18],[20]. Cleavage of CD43 and CD45 by StcE on the neutrophil surface, leading to increased adhesion, defective migration, increased respiratory burst production, and overall dysregulation of the immune response, may provide another mechanism by which StcE enhances the progression of disease caused by enterohemorrhagic *E. coli*. Moreover, as CD43 and CD45 are expressed on many cells of the immune system, it is unlikely that the immunomodulatory activity of StcE is limited to neutrophils.

## Materials and Methods

### Ethics Statement

For studies with neutrophils from human subjects, informed consent was obtained from healthy donors at the time of blood draw with approval of the University of Wisconsin-Madison Center for Health Sciences Human Subjects committee.

### Reagents

Recombinant StcE and E435D protein were expressed and purified as described [20]. Endotoxin was removed using Endotrap blue columns (Lonza) according to manufacturer's instructions. Samples were evaluated by Lonza Endotoxin Testing Services, and endotoxin levels were routinely <1 EU/mL. Dulbecco's Phosphate-Buffered Saline with Ca^2+^/Mg^2+^ (DPBS+/+, Mediatech) of an equivalent volume to soluble protein was used as a vehicle control. Heat-inactivated StcE (hiStcE) was produced by incubation at 65°C for 10 minutes (Grys 2006). Interleukin-8 (IL-8), f-Met-Leu-Phe (fMLP), fibrinogen (Fbg), bovine serum albumin (BSA), and phorbol myristate acetate (PMA) were purchased from Sigma. Human serum albumin (HSA) was from ZLB Bioplasma AG (Berne, Switzerland). Phycoerythrin (PE)-conjugated anti-CD43 clone L10 and FITC-conjugated anti-CD45 clone HI30 were obtained from Caltag (Invitrogen). The anti-CD43 C-terminal domain antibody (sc-7052) was obtained from Santa Cruz Biotechnology. PE-conjugated anti-CD45RO clone UCHL1 was obtained from Ebioscience (San Diego, CA). EM-grade 16% paraformaldehyde (PFA) was from Electron Microscopy Sciences (Hartfield, PA) and 16% formaldehyde was from Polysciences (Warrington, PA).

### Primary Cells and Cell Lines

Peripheral blood neutrophils were purified from human blood using Polymorphprep according to manufacturer's recommendations (Nycomed, Sheldon, UK). HL-60 cells (ATCC) were maintained in Iscove's Modified Dulbecco's Medium (IMDM) according to ATCC guidelines. HL-60 cells were differentiated (dHL-60s) as previously described [48]. Primary human lung microvascular endothelial cells (HMVEC-L) were maintained in EGM-2MV media according to manufacturer's instructions (Lonza). For transmigration assays, cells were seeded on collagen-coated 3 µm pore, 0.33 cm^2^ polycarbonate transwell inserts (Costar) at a density of 1×10^5^ cells.

### Western Blotting and Immunoprecipitation

For cleavage reactions, 1×10^6^ dHL-60s in IMDM were treated with 1 µg/mL StcE, E435D or vehicle control for 30 min at 37°C, 5% CO_2_, followed by separation of supernatants and cell pellets. Supernatants were precipitated with 10% trichloracetic acid (TCA) on ice. Samples were separated by SDS-PAGE, transferred to nitrocellulose or PVDF and immunoblotted using standard methods [49]. L10 and HI-30 antibodies were used at 1∶500 and sc-7052 was used at 1∶200. Samples were detected by enhanced chemoluminescence (ECL) using Immobilon HRP substrate (Millipore). For direct precipitation of binding partners, StcE was expressed with an uncleavable intein-chitin binding domain fusion tag (CBD) in vector pTYB11 and purified using the IMPACT system as previously described [20]. Purified StcE-CBD bound to chitin beads (CB) was incubated with 1×10^7^ lysed dHL-60s. CB alone served as a negative control, and samples were processed using standard methods for co-immunoprecipitations [49].

### Flow Cytometry

Neutrophils (1×10^6^/mL) were treated with 1 µg/mL StcE or E435D (unless otherwise indicated) or vehicle control in EGM-2MV for 30 min at 37°C, 5% CO_2_. Cells were blocked in DPBS+/− with 0.05% BSA and 0.01% HSA and incubated with primary antibody per manufacturer instructions. Samples were read using an LSRII flow cytometer (Becton Dickinson). For the flow oxidative burst (OB) assay, neutrophils (2.5×10^6^) were labeled with dihydrorhodamine 123 (DHR) (Molecular Probes) as described [50],[51] and treated with 5 µg/mL StcE or E435D, vehicle control, or 30 ng/mL PMA for 30 min at 37°C, 7.5% CO_2_. Oxidation by H_2_O_2_ and O_2_^−^ of DHR to rhodamine was measured as fluorescence of live cells in the FITC channel. Data were analyzed using FlowJo (Treestar) and individual values from five independent experiments were combined and analyzed by one-way ANOVA using GraphPad Prism.

### Immunofluorescent Staining

Neutrophils (1×10^5^/mL) in EGM-2MV were plated on Fbg-coated (10 µg/mL) glass coverslips for 30 min at 37°C, 5% CO_2_ in the presence of 20 µg/mL StcE, E435D or vehicle control, and further incubated for 10 min in the presence of 100 nM fMLP. Samples were fixed in 1% PFA in DPBS, post-fixed in 1% formic acid, and permeabilized in 0.1% Triton-X 100 (Sigma). Primary antibodies were used at 1∶200 (anti-StcE), 1∶100 (L10 and HI30), or 1∶25 (sc-7052). Goat anti-rabbit Alexa Fluor 633, goat anti-mouse Alexa Fluor 488, and rabbit anti-goat Alexa Fluor 488 secondary antibodies were used at 1∶200, and samples were mounted in ProLong Gold antifade reagent with DAPI (Molecular Probes). Coverslips were imaged using a 63× oil immersion lens on a Zeiss LSM510 confocal microscope. Data were obtained and analyzed using LSM 5 Image Software (Zeiss).

### Video Microscopy

Non-tissue culture-treated dishes were coated with 10 µg/mL Fbg, and 5×10^5^ neutrophils were plated in the presence of 8.33 µg/mL StcE or E435D or vehicle control in EGM-2MV for 30 min at 37°C, 7.5% CO_2_. 1.25 nM IL-8 or 100 nM fMLP was included for the last 5 or 10 min as indicated. Dishes were placed in The Box closed system (Life Imaging Services, Reinach Switzerland) at 37°C and imaged on an Olympus IX-70 inverted microscope (Olympus America) using a 20× phase objective. Images were collected using a Coolsnap *fx* cooled charged-coupled device (CCD) video camera (Photometrics, Huntington Beach, CA) and captured into Metaview v6.2 (Universal Imaging Corp., Downingtown, PA) every 15 s for 10 min. To obtain measurements of cell velocity, cell centroids were tracked for the first 21 frames using MetaMorph v7.0r2. For quantitation of cell length, all cells in two random frames were measured using MetaMorph. Means of at least three independent experiments were combined and analyzed by one-way ANOVA using Prism with Dunnett posttest.

### Adhesion Assay

Neutrophils were fluorescently labeled as described [52] and brought to 2×10^6^/mL in EGM-2MV. 50 µL of cell suspension were added to StcE, E435D, hiStcE, or vehicle control serially diluted in 50 µL EGM-2MV in a Fbg-coated 96-well black plate (Greiner, Kremsmuenster, Upper Austria). Cells were allowed to adhere for 40 min at 37°C, 7.5% CO_2_. Positive control cells were treated with 100 nM fMLP for the final 10 min. Samples were washed 3 times and the fluorescence of remaining adherent cells measured using a Gemini EM microplate spectrofluorometer (Molecular Devices) with excitation/emission at 485/530 nm. A standard curve was included on each plate, and linear regression was performed with Prism to determine number of neutrophils adhered in each well. Relative adhesion was calculated by normalizing the number of adherent cells to the average values for vehicle control in the absence of fMLP. Means of at least three independent experiments performed in duplicate were combined and analyzed by two-way analysis of variance (ANOVA) using Prism with Bonferroni post test.

### Transwell Assay

Neutrophil migration was determined using transwell assays essentially as described (Lokuta 2005). 3 µm transwell filters were coated with 2.5 µg/mL Fbg or a monolayer of HMVEC-L, and 4×10^5^ calcein-AM-labeled neutrophils were placed in the top chamber with 25 µg/mL (filters) or 50 µg/mL (monolayers) StcE, E435D, hiStcE, or vehicle control. EGM-2MV alone or containing 100 nM fMLP was placed in the bottom chamber and samples were incubated at 37°C, 5% CO_2_ for 210 min. 50 mM EDTA was added to the lower chamber, transwells were removed and the fluorescence of migrated cells quantitated as described for adhesion assays. Numbers of transmigrated cells were normalized to vehicle control with chemoattractant, and means of at least three independent experiments were combined and analyzed by one-way ANOVA using Prism with Bonferroni post test.

### Zebrafish

Zebrafish were bred and maintained as previously described (Mathias 2006). At 3 days post fertilization (dpf), zebrafish embryos were anesthetized in 3 mL embryo water (E3) containing 0.1 mg/mL tricaine and 25 µg/mL StcE, hiStcE, or vehicle control. Zebrafish were wounded in the dorsal tail fin with the tip of a 25 gauge needle, and the bath replaced with protein treatment in E3 without tricaine. After 24 hours at 28°C, zebrafish were fixed and stained for whole-mount immunofluorescence as previously described (Mathias 2006). Rabbit polyclonal anti-myeloperoxidase antibody and goat anti-rabbit Alexa Fluor 488 (Molecular Probes) were used at 1∶500. Images were acquired with a Nikon SMZ-1500 zoom microscope with epifluorescent illumination using MetaMorph software. For quantitation of inflammation, images of individual fish were compiled and blinded, and the number of neutrophils in the dorsal and ventral fin caudal to the yolk sac were counted. Means of four independent experiments were combined and analyzed by one-way ANOVA using Prism with Dunnett's post test.

### Accession Numbers

Swissprot ID numbers for proteins described in the text are as follows: StcE (O82882); CD43 (P16150), CD45 (P08575).

## Supporting Information

Figure S1 Direct precipitation of neutrophil lysates with StcE. Lysates of dHL-60s or primary neutrophils (1×10^7^)were incubated in the presence of EDTA with StcE crosslinked to agarose beads (Affigel 15, Biorad, Hercules, CA). Reactions were separated by SDS-PAGE, and stained with ProQ Emerald 300 glycoprotein staining kit (Molecular Probes) or Sypro Ruby total protein staining kit (not shown). Beads alone were included as a control for background staining. Glycoprotein bands of molecular weight consistent with CD43 and CD45 are indicated by arrows. An indistinct band of ∼100 kDa (indicated by unlabeled arrow) was present in pulldowns of dHL-60, but not neutrophil, lysates. This may be an aberrantly expressed glycoprotein that reflects the leukemic origin of HL-60s; similar results were obtained with Jurkat T cells (unpublished data).
(0.11 MB PDF)

Figure S2 StcE does not bind appreciably to fibrinogen. ELISA plates were coated with increasing concentrations of Fbg, incubated with varying concentrations of StcE, and detected with a polyclonal antibody to StcE followed by goat anti-rabbit conjugated to horseradish peroxidase. Reactions were developed using TMB substrate kit (Pierce) and measured spectrofluorometrically. Data shown are from a representative of three independent experiments performed in duplicate.
(0.05 MB PDF)

Figure S3 Competitive binding of E435D to the extracellular domain of CD43. Neutrophils (1×10^6^) were treated with varying concentrations of E435D for 30 min at 37°C, 5% CO_2_. Cells were stained with L10-PE and analyzed by flow cytometry to detect masking of the L10 epitope by E435D as compared to vehicle control. Geometric mean fluorescence intensity is also shown for each sample, and data shown are representative of three independent experiments. StcE treatment served as a positive control for removal of the L10 epitope, and cleavage was far more efficient than binding of E435D at reducing L10 staining. Staining for total CD45 with the HI30 mAb, which recognizes an epitope that is unaffected by StcE cleavage, served as a negative control for epitope blocking. E435D did not affect the binding of HI30.
(0.12 MB PDF)

Video S1 Random migration of HSA-treated neutrophils. For all videos, PMN were treated with 12.5 µg/mL protein for 30 min at 37°C, 7.5% CO_2_. Time lapse images were collected over 10 min at 15 s intervals.
(1.72 MB AVI)

Video S2 Random migration of StcE-treated neutrophils.
(1.75 MB AVI)

Video S3 Random migration of E435D mutant-treated neutrophils.
(1.76 MB AVI)

Video S4 Random migration of HSA-treated neutrophils stimulated with IL-8.
(1.72 MB AVI)

Video S5 Random migration of StcE-treated neutrophils stimulated with IL-8.
(1.75 MB AVI)

Video S6 Random migration of E435D mutant-treated neutrophils stimulated with IL-8.
(1.75 MB AVI)
