# Supplementary figures and images for: Modulation of Neutrophil Function by a Secreted Mucinase of Escherichia coli O157∶H7
Source: PLoS Pathog. 2009 Feb 27;5(2):e1000320. doi: 10.1371/journal.ppat.1000320 (PMC2642718; doi:10.1371/journal.ppat.1000320)

dHL-60

Neutrophil

Beads

CD45 →

CD43 →

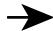

75 kDa

25 kDa

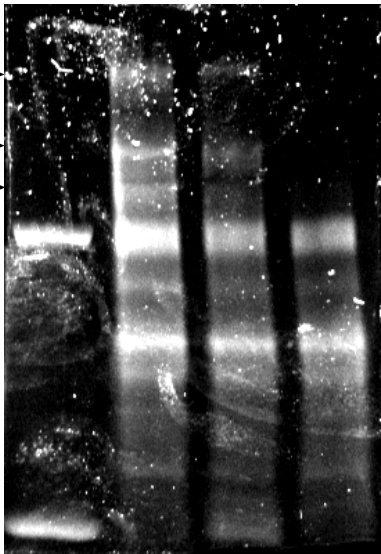

Supplement: Figure S1 — Direct precipitation of neutrophil lysates with StcE. Lysates of dHL-60s or primary neutrophils (1×10^7^)were incubated in the presence of EDTA with StcE crosslinked to agarose beads (Affigel 15, Biorad, Hercules, CA). Reactions were separated by SDS-PAGE, and stained with ProQ Emerald 300 glycoprotein staining kit (Molecular Probes) or Sypro Ruby total protein staining kit (not shown). Beads alone were included as a control for background staining. Glycoprotein bands of molecular weight consistent with CD43 and CD45 are indicated by arrows. An indistinct band of ∼100 kDa (indicated by unlabeled arrow) was present in pulldowns of dHL-60, but not neutrophil, lysates. This may be an aberrantly expressed glycoprotein that reflects the leukemic origin of HL-60s; similar results were obtained with Jurkat T cells (unpublished data). (0.11 MB PDF) [file ppat.1000320.s001.pdf]

# ELISA for Binding of StcE to Fibrinogen

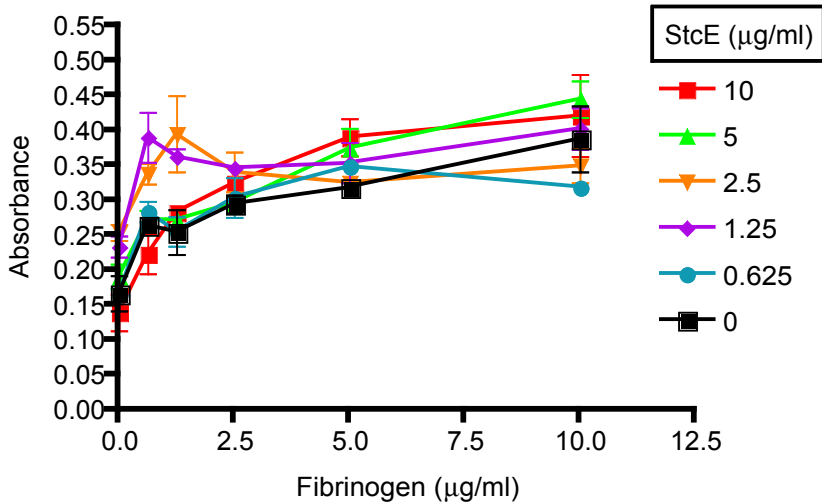

Supplement: Figure S2 — StcE does not bind appreciably to fibrinogen. ELISA plates were coated with increasing concentrations of Fbg, incubated with varying concentrations of StcE, and detected with a polyclonal antibody to StcE followed by goat anti-rabbit conjugated to horseradish peroxidase. Reactions were developed using TMB substrate kit (Pierce) and measured spectrofluorometrically. Data shown are from a representative of three independent experiments performed in duplicate. (0.05 MB PDF) [file ppat.1000320.s002.pdf]
