# Supplementary material for: Modulation of Neutrophil Function by a Secreted Mucinase of Escherichia coli O157∶H7
Source: PLoS Pathog. 2009 Feb 27;5(2):e1000320. doi: 10.1371/journal.ppat.1000320 (PMC2642718; doi:10.1371/journal.ppat.1000320)
Supplement: Figure S3 — Competitive binding of E435D to the extracellular domain of CD43. Neutrophils (1×10^6^) were treated with varying concentrations of E435D for 30 min at 37°C, 5% CO2. Cells were stained with L10-PE and analyzed by flow cytometry to detect masking of the L10 epitope by E435D as compared to vehicle control. Geometric mean fluorescence intensity is also shown for each sample, and data shown are representative of three independent experiments. StcE treatment served as a positive control for removal of the L10 epitope, and cleavage was far more efficient than binding of E435D at reducing L10 staining. Staining for total CD45 with the HI30 mAb, which recognizes an epitope that is unaffected by StcE cleavage, served as a negative control for epitope blocking. E435D did not affect the binding of HI30. (0.12 MB PDF) [file ppat.1000320.s003.pdf]

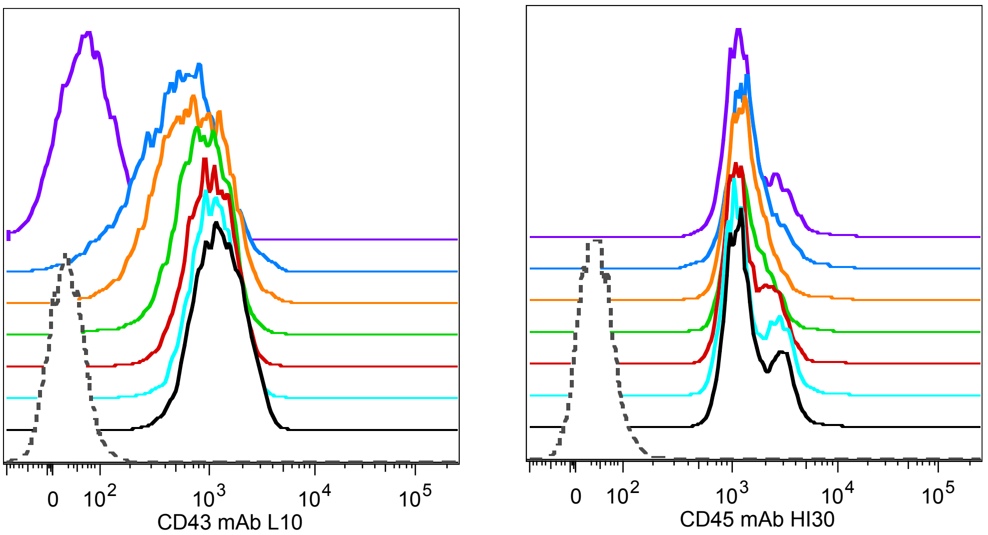


| | Protein Concentration (µg/mL) | Geometric Mean Fluorescence CD43 | Geometric Mean Fluorescence CD45 |
|---|---|---|---|
| unstained | | 23 | 35 |
| vehicle | 0 | 1169 | 1406 |
| E435D | 0.5 | 1028 | 1407 |
| E435D | 5 | 959 | 1355 |
| E435D | 12.5 | 787 | 1331 |
| E435D | 25 | 641 | 1334 |
| E435D | 50 | 492 | 1439 |
| StcE | 2 | 67 | 1370 |
